# Reading the mind in the nose

**DOI:** 10.1177/20416695231163449

**Published:** 2023-03-20

**Authors:** Maximilian Davide Broda, Benjamin de Haas

**Affiliations:** Experimental Psychology, 9175 Justus Liebig University Giessen, Germany;; Center for Mind, Brain and Behavior (CMBB), University of Marburg and Justus Liebig University Giessen, Germany

**Keywords:** emotion, face perception, features/parts, perception, nose

## Abstract

Humans infer mental states and traits from faces and their expressions. Previous research focused on the role of eyes and mouths in this process, even though most observers fixate somewhere in between. Here, we report that ratings of the nose region are surprisingly consistent with those for the full face and even with subjective feelings of the nose bearer. We propose the nose as central to faces and their perception.

Faces tell us a lot about their owners—or at least we like to believe they do (Todorov, 2017). We infer dominance, trustworthiness, attractiveness and emotional states from facial looks. Which parts of the face do we use for such sweeping conclusions? Most research has focused on the eye and mouth regions (e.g., Kim et al., 2022), even though average and expert face observers tend to look somewhere *in between* (Linka et al., 2022; Peterson & Eckstein, 2012). In fact, we ourselves plead guilty to this oversight (Broda & de Haas, 2022a, 2022b; de Haas et al., 2016, 2021; de Haas & Schwarzkopf, 2018). But there is more to faces. Following intensive examination of ourselves and others, we discovered: Noses. Noses are as individually different as the traits and states of their wearers. They exist in all shapes and sizes, can smell and even run.

Here, we report that participants can rate expressions and traits from isolated nose regions. In an online experiment, 114 participants rated isolated nose, eye and mouth regions of 30 frontal face images that were taken from the fLoc functional localizer package (Stigliani et al., 2015). All participants gave informed consent and were compensated with course credits. The study was approved by the institutional review board and in accordance with the declaration of Helsinki. Ratings of face parts proceeded in random order and along five dimensions. This was followed by ratings of the corresponding full-face images. We correlated the average rating of each face part with that for the corresponding full-face ratings in turn. As can be seen in Figure 1a and b, nasal ratings showed significant consistency with those for full faces (all *R*^2 ^= 20–54%). The extreme examples shown for each dimension may serve to convince the reader of the expressive power of the nose. While we fully concede that some of this effect may be due to parts of the region outside nose proper (such as the cheek), we maintain its expressive force may extend to the nose itself. Consider Figure 1c, which shows the results of a ground truth validation. The senior author employed a method acting approach to enter states of low or high valence by imagining desk rejection or immediate acceptance of this manuscript. This intervention led to remarkable engagement of the central face region, clearly including the nose itself. Observers of the nose were indeed able to track the underlying mental states, as reflected in the stark difference of their valence ratings for the positive *vs*. negative expression. Readers taking a liking towards null hypothesis significance testing (Cohen, 1994) may be excited to learn that MATLAB 2021a (Natick, MA) was incapable of computing the precise *p*-value for the corresponding paired *t*-test (*t* > .20) because *p* was too low. The consistency for eyes and mouths tended to be higher than for noses (though this difference failed to reach statistical significance in most cases, probably due to limited power; Table S1). Interestingly, nose consistencies were higher for valence, arousal, and attractiveness than the two personality dimensions. Previous research indicates valence can serve as a proxy for trustworthiness (Oosterhof & Todorov, 2008). Indeed, valence and trustworthiness ratings were correlated for full faces (*r* = .78, *p* < .001), mouths (*r* = .79, *p* < .001), eyes (*r* = .67, *p* < .001), and noses (*r* = .77, *p* < .001). Overall, we conclude that nose regions convey a surprising degree of information about their bearers.

**Figure 1.**
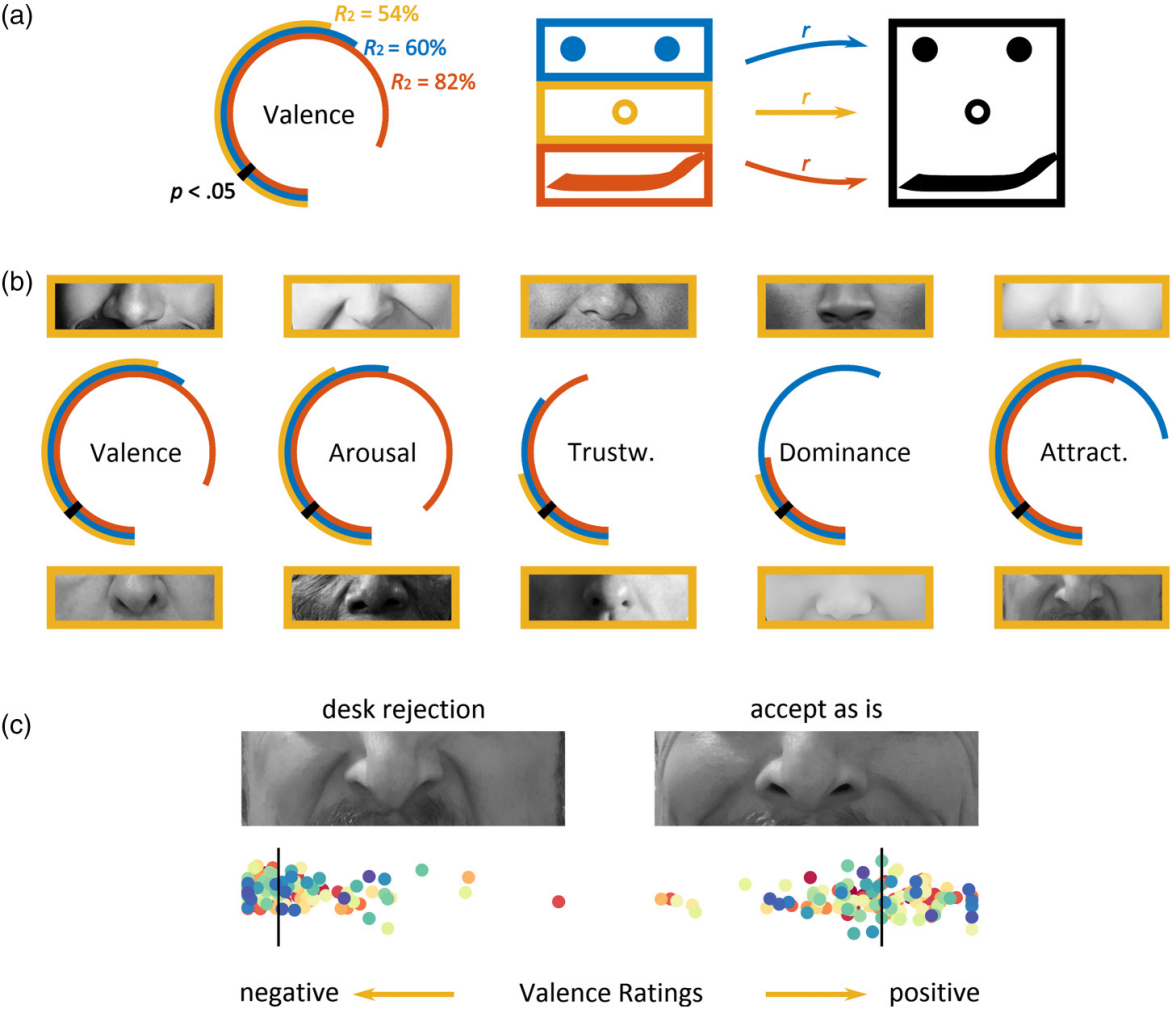
Consistent and valid perception of nasal traits and states. (a) Analysis approach and illustration of ring plots. We correlated ratings for isolated face regions with corresponding ratings for the respective whole faces. Nested ring plots show the proportion of variance in whole-face ratings explained by feature ratings. Variance explained by nose, eye, and mouth ratings is shown as the outer to the innermost ring in yellow, blue, and red, respectively. The effect size corresponding to a *p*-value of .05 is marked in black. (b) Consistency of part-based ratings of valence, arousal, trustworthiness, dominance, and attractiveness with those for full-face images. Nasal images in the upper and lower row show extreme examples of stimuli rated high and low along each dimension. The proportion of full-face variance explained by nose-, eye- and mouth-based ratings, respectively, were 54%, 60% and 82% for valence; 43%, 53% and 88% for arousal; 21%, 36% and 46% for trustworthiness; 21%, 57% and 24% for dominance; 50%, 72% and 57% for attractiveness. (c) Validity of nasal valence ratings. The senior author imagined immediate desk rejection or acceptance of this manuscript to enter states of low and high valence, respectively. The corresponding nasal ratings confirm observers’ ability to read the mind in the nose. Each scatter point corresponds to the rating of one observer, with the left border of either image corresponding to *feeling extremely negative* and the right border to *feeling extremely positive*. Black lines correspond to median ratings across observers. Data, code and materials can be found at https://osf.io/npehx/.

## Supplemental Material

sj-docx-1-ipe-10.1177_20416695231163449 - Supplemental material for Reading the mind in the nose Click here for additional data file. Supplemental material, sj-docx-1-ipe-10.1177_20416695231163449 for Reading the mind in the nose by Maximilian Davide Broda and Benjamin de Haas in i-Perception
