# Supplementary material for: Reading the mind in the nose
Source: Iperception. 2023 Mar 20;14(2):20416695231163449. doi: 10.1177/20416695231163449 (PMC10028657; doi:10.1177/20416695231163449)
Supplement: sj-docx-1-ipe-10.1177_20416695231163449 - Supplemental material for Reading the mind in the nose [file sj-docx-1-ipe-10.1177_20416695231163449.docx]

**Supplemental Material**

Table S1 Steiger-Z test and uncorrected p-values for comparisons of whole-face consistencies between features for each dimension.

|  | **Valence** | **Arousal** | **Trustw.** | **Dominance** | **Attractive.** |
| --- | --- | --- | --- | --- | --- |
| **Eyes vs. Nose** | 0.35, *p*=.723 | 0.49, *p*=.625 | 0.72, *p*=.473 | 1.77, *p*=.077 | 1.87, *p*=.061 |
| **Eyes vs. Mouth** | -1.69, *p*=.091 | -2.53, *p*=.011 | -0.44, *p*=.659 | 1.50, *p*=.133 | 1.11, *p*=.269 |
| **Mouth vs. Nose** | 2.24, *p*=.025 | 3.89, *p*<.001 | 1.36, *p*=.173 | 0.15, *p*=.884 | 0.41, *p*=.680 |
